# Supplementary material for: A Molecular Hybrid of the GFP Chromophore and 2,2′-Bipyridine: An Accessible Sensor for Zn2+ Detection with Fluorescence Microscopy
Source: Int J Mol Sci. 2024 Mar 20;25(6):3504. doi: 10.3390/ijms25063504 (PMC10971390; doi:10.3390/ijms25063504)
Supplement: Supplementary file 1 [file ijms-25-03504-s001.zip › ijms-2898390-supplementary.pdf]

Supplementary information for

A molecular hybrid of the GFP chromophore and 2,2′-bipyridine: an accessible sensor for Zn^{2+} detection with fluorescence microscopy

Attila Csomos[1,2], Miklós Madarász[3], Gábor Turczel[4], Levente Cseri[3,5], Gergely Katona,[6] Balázs J. Rózsa,[3,6,7,*] Ervin Kovács,[6,8,*] and Zoltán Mucsi[1,3,9]

1 Department of Chemistry, Femtonics Ltd., Tűzoltó utca 59, H-1094 Budapest, Hungary
2 Hevesy György PhD School of Chemistry, Eötvös Loránd University, Pázmány Péter sétány 1/A, H-1117 Budapest, Hungary
3 BrainVisionCenter, Liliom utca 43, H-1094 Budapest, Hungary
4 NMR Research Laboratory, HUN-REN Research Centre for Natural Sciences, Magyar Tudósok körútja 2, H-1117 Budapest
5 Department of Organic Chemistry & Technology, Budapest University of Technology & Economics, 3. Muegyetem rakpart, H-1111 Budapest, Hungary Budapest, Hungary
6 Two-Photon Measurement Technology Research Group, Pázmány Péter Catholic University, Práter u. 50/a, H-1083 Budapest, Hungary
7 Laboratory of 3D Functional Network and Dendritic Imaging, Institute of Experimental Medicine, Szigony u. 43, H-1083 Budapest, Hungary
8 Polymer Chemistry and Physics Research Group, HUN-REN Research Centre for Natural Sciences, Magyar Tudósok körútja 2, H-1117 Budapest, Hungary
9 Faculty of Materials and Chemical Sciences, University of Miskolc, H-3515 Miskolc, Hungary

* Correspondence: kovacs.ervin@ttk.hu (E.K.); zoltan.mucsi@uni-miskolc.hu (Z.M.)

Table of Contents

1. NMR and HRMS spectra of GFZnP BIPY 2
2. Additional spectroscopic data 8
3. Additional biological data 10
4. Theoretical studies 13
4.1 Raw computational data 16
4.2 The coordinates of computed geometries 17

1. NMR and HRMS spectra of GFZnP BIPY

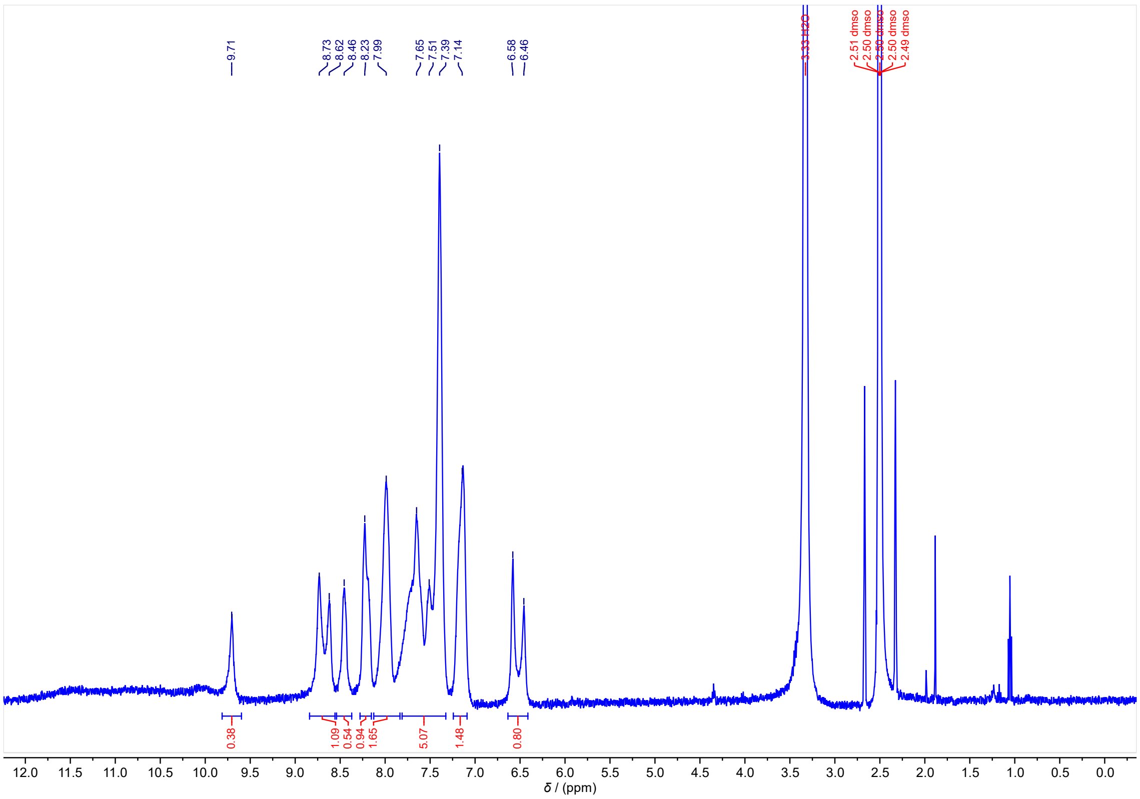

Fig. S1. ^{1}H NMR spectrum of GFZnP BIPY complex recorded at 400 MHz in DMSO-*d6.*

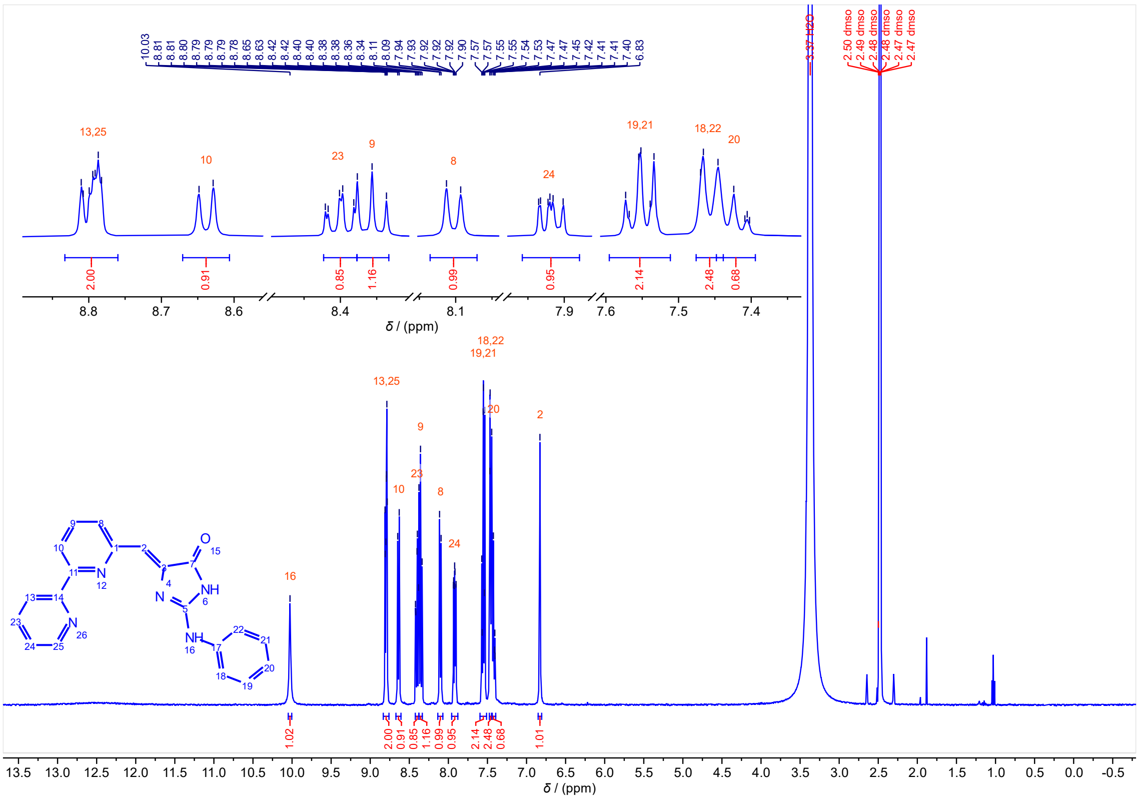

Fig. S2. ^{1}H NMR spectrum of GFZnP BIPY - Zn^{2+} complex recorded at 400 MHz in DMSO-*d6.*

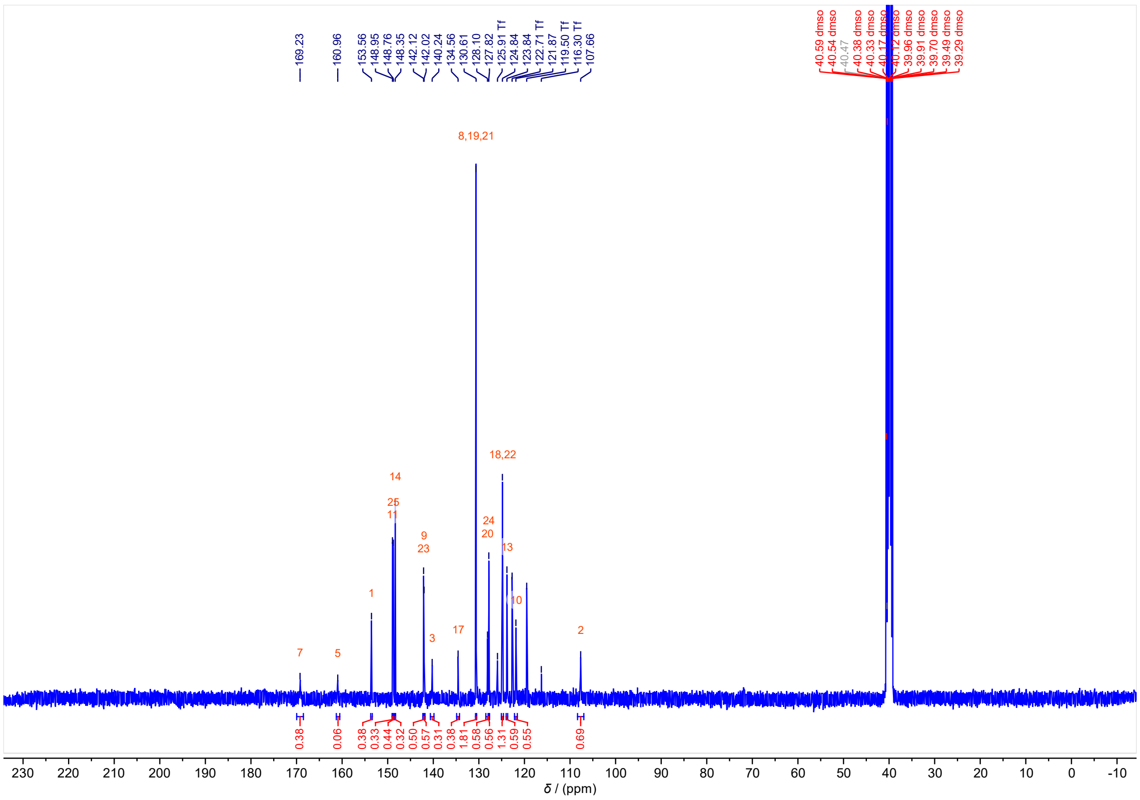

Fig. S3. ^{13}C NMR spectrum of GFZnP BIPY - Zn^{2+} complex recorded at 101 MHz in DMSO-*d6*.

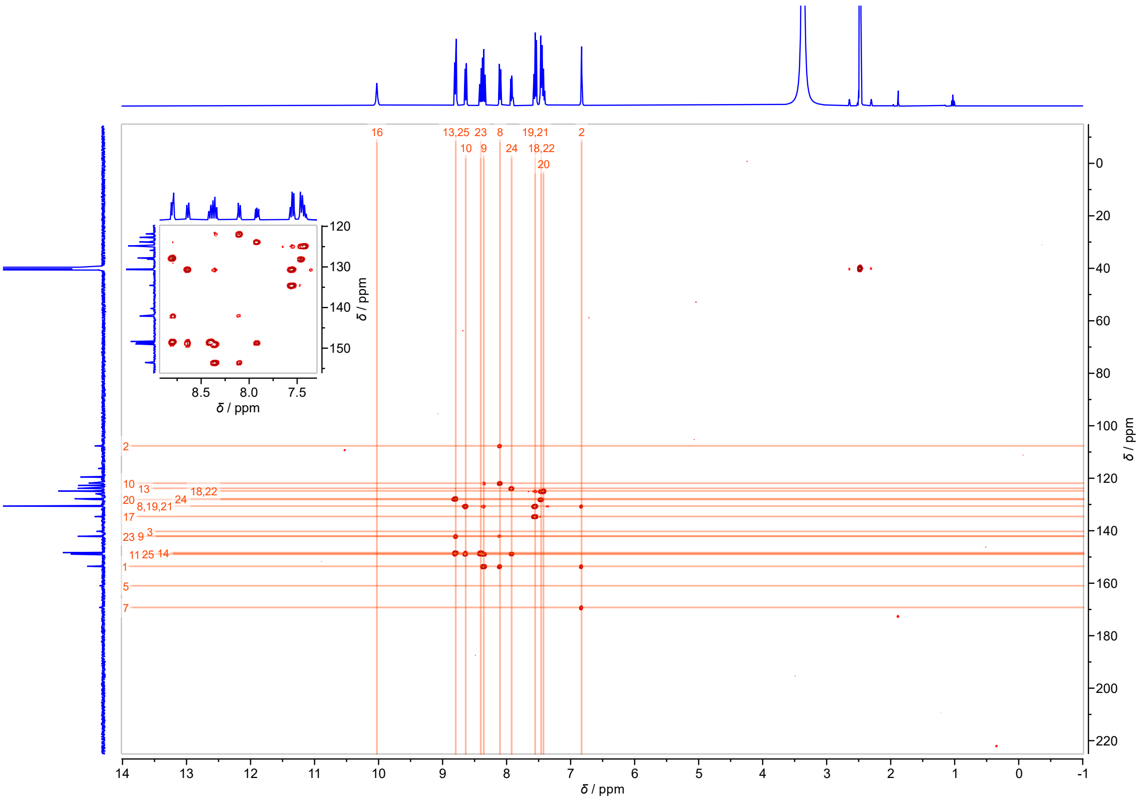

Fig. S4. HSQC spectrum of GFZnP BIPY - Zn^{2+} complex recorded at 400 MHz in DMSO-*d6*.

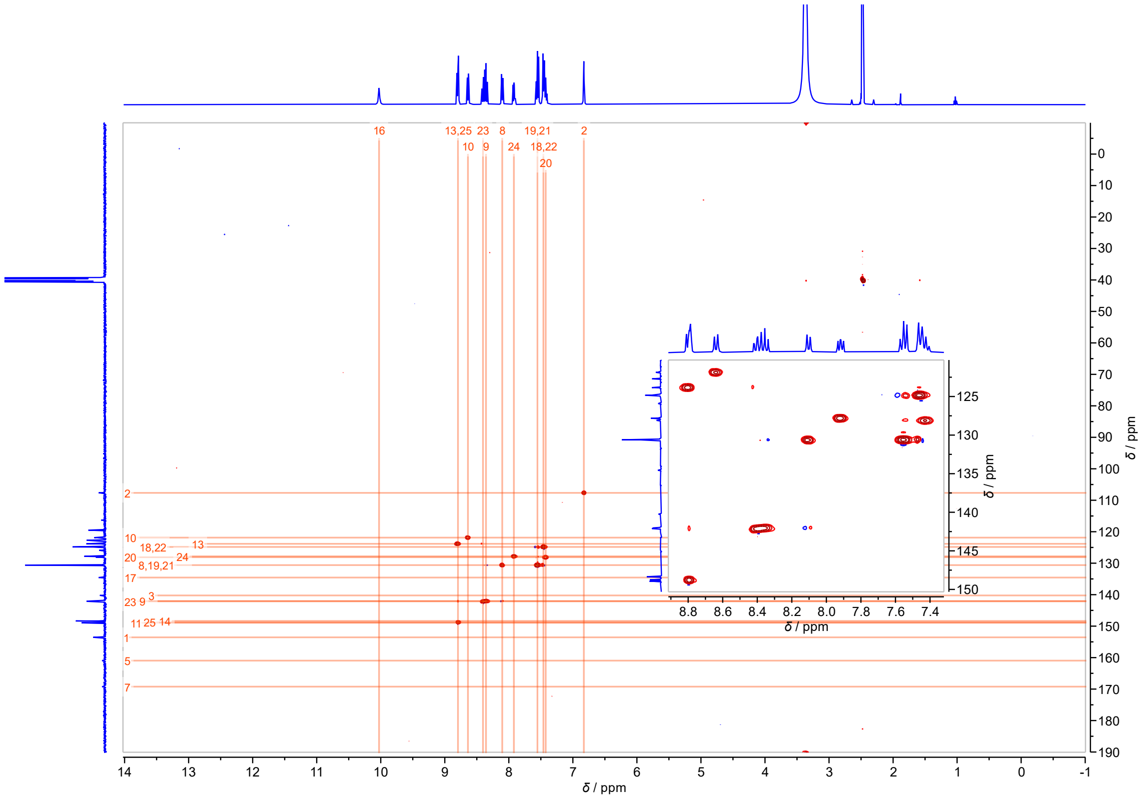

Fig. S5. HMBC spectrum of GFZnP BIPY - Zn^{2+} complex recorded at 400 MHz in DMSO-*d6*.

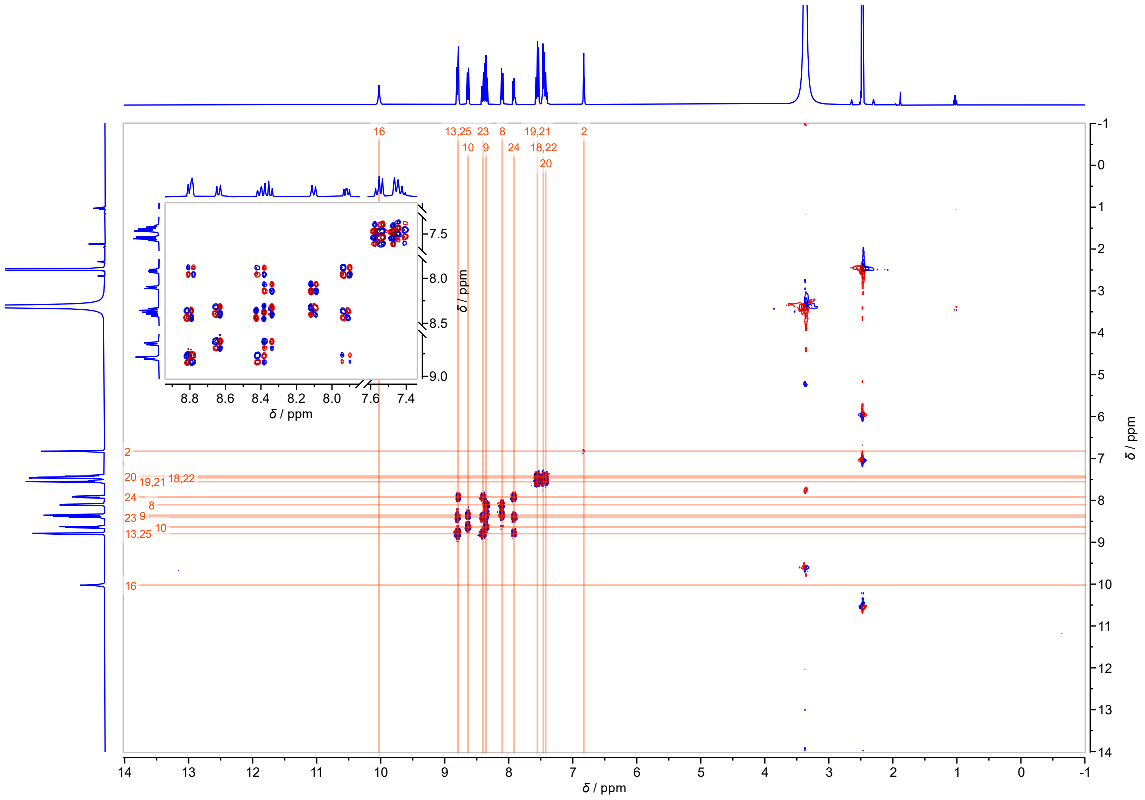

Fig. S6. COSY spectrum of GFZnP BIPY - Zn^{2+} complex recorded at 400 MHz in DMSO-*d6*.

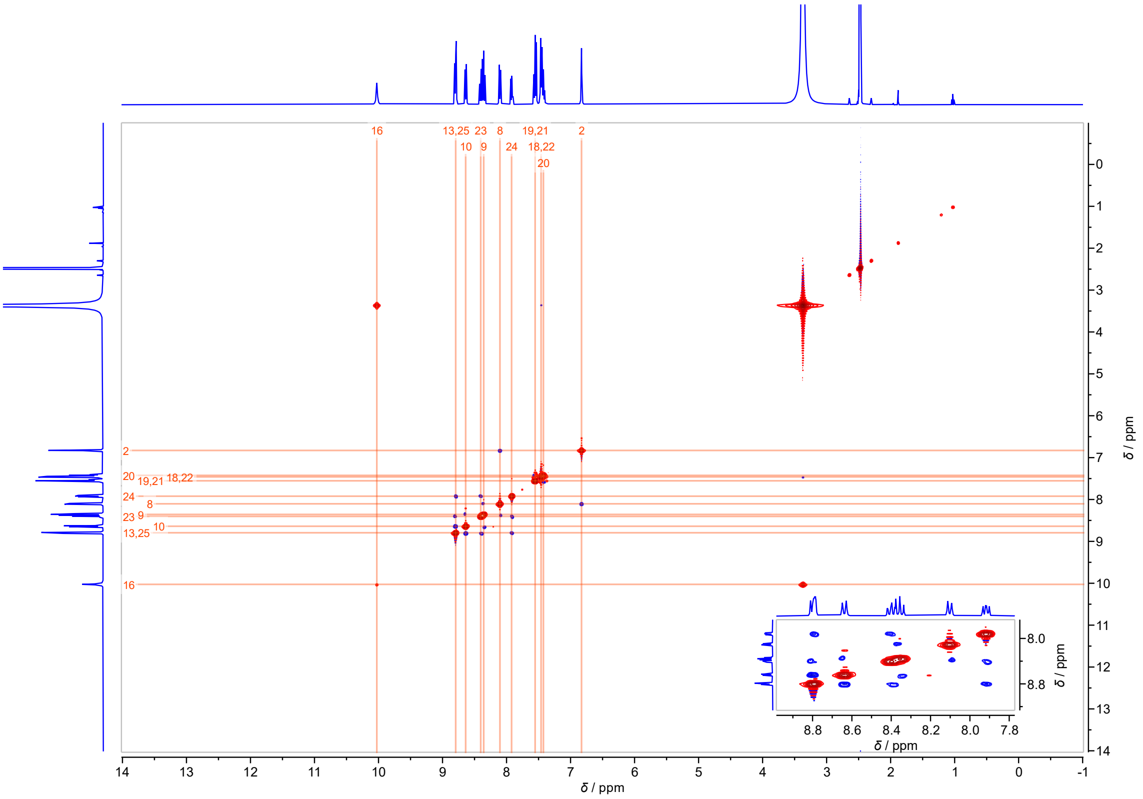

Fig. S7. ROESY spectrum of GFZnP BIPY - Zn^{2+} complex recorded at 400 MHz in DMSO-*d6*.

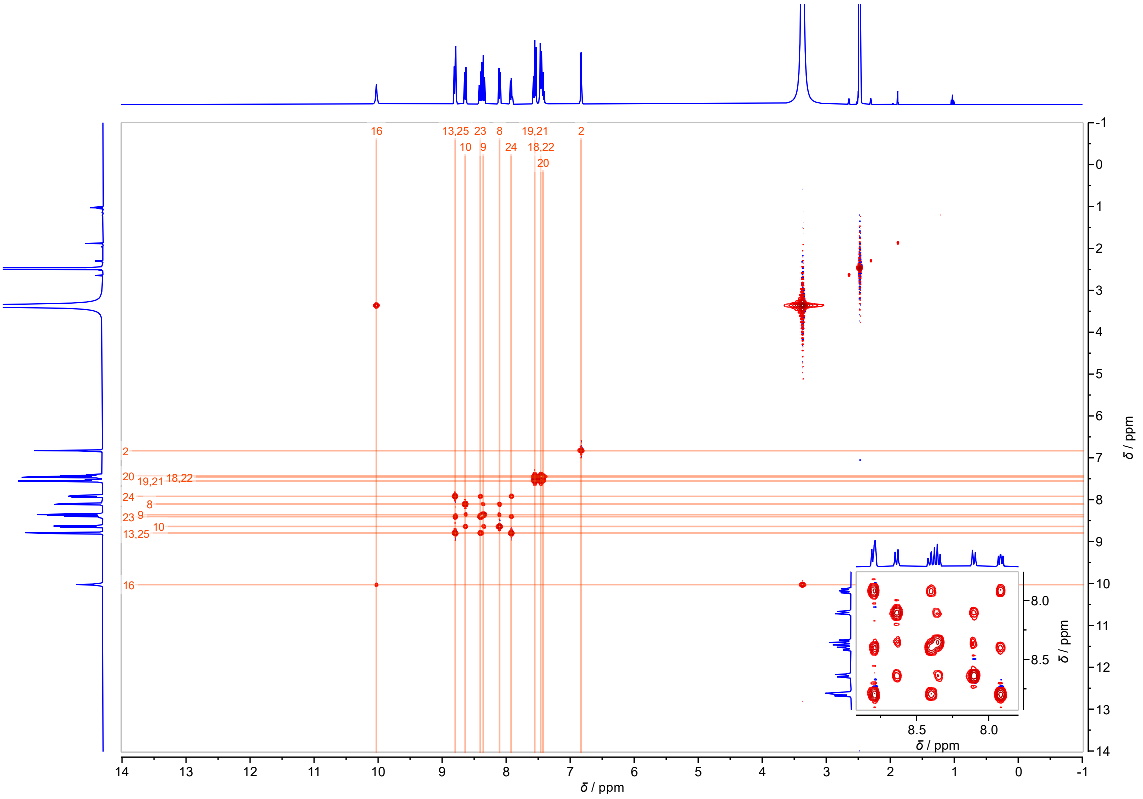

Fig. S8. TOCSY spectrum of GFZnP BIPY - Zn^{2+} complex recorded at 400 MHz in DMSO-*d6*.

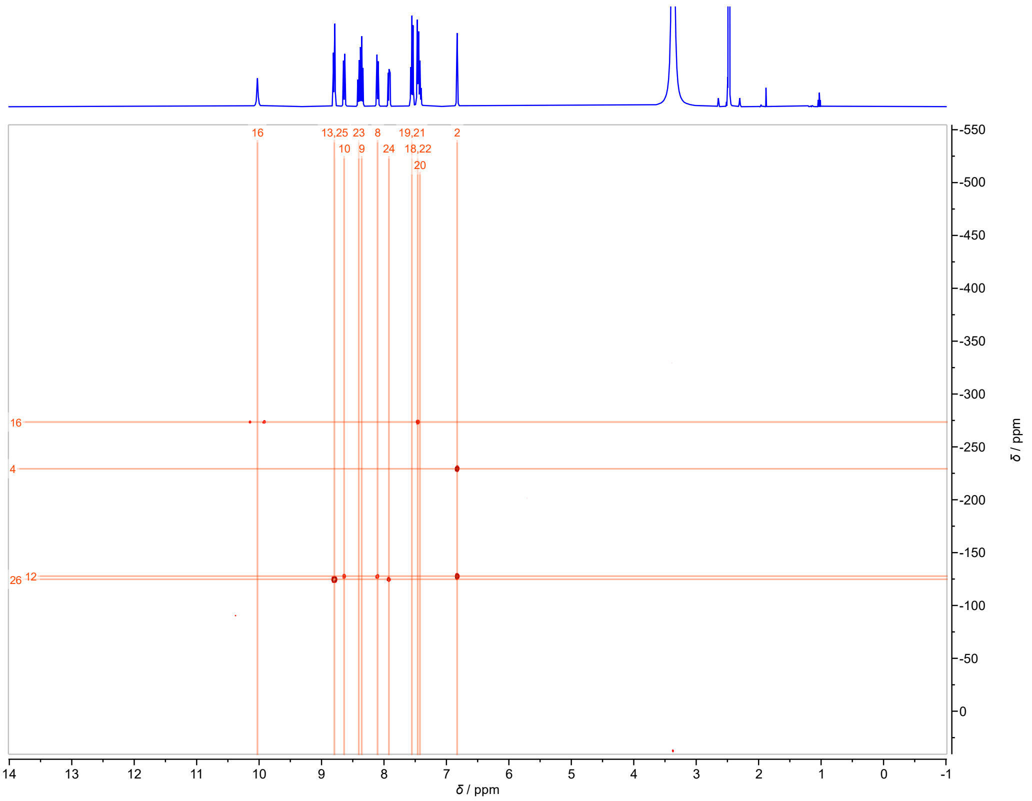

Fig. S9. ^{15}N HMBC spectrum of GFZnP BIPY - Zn^{2+} complex recorded at 41 MHz in DMSO-*d6.*

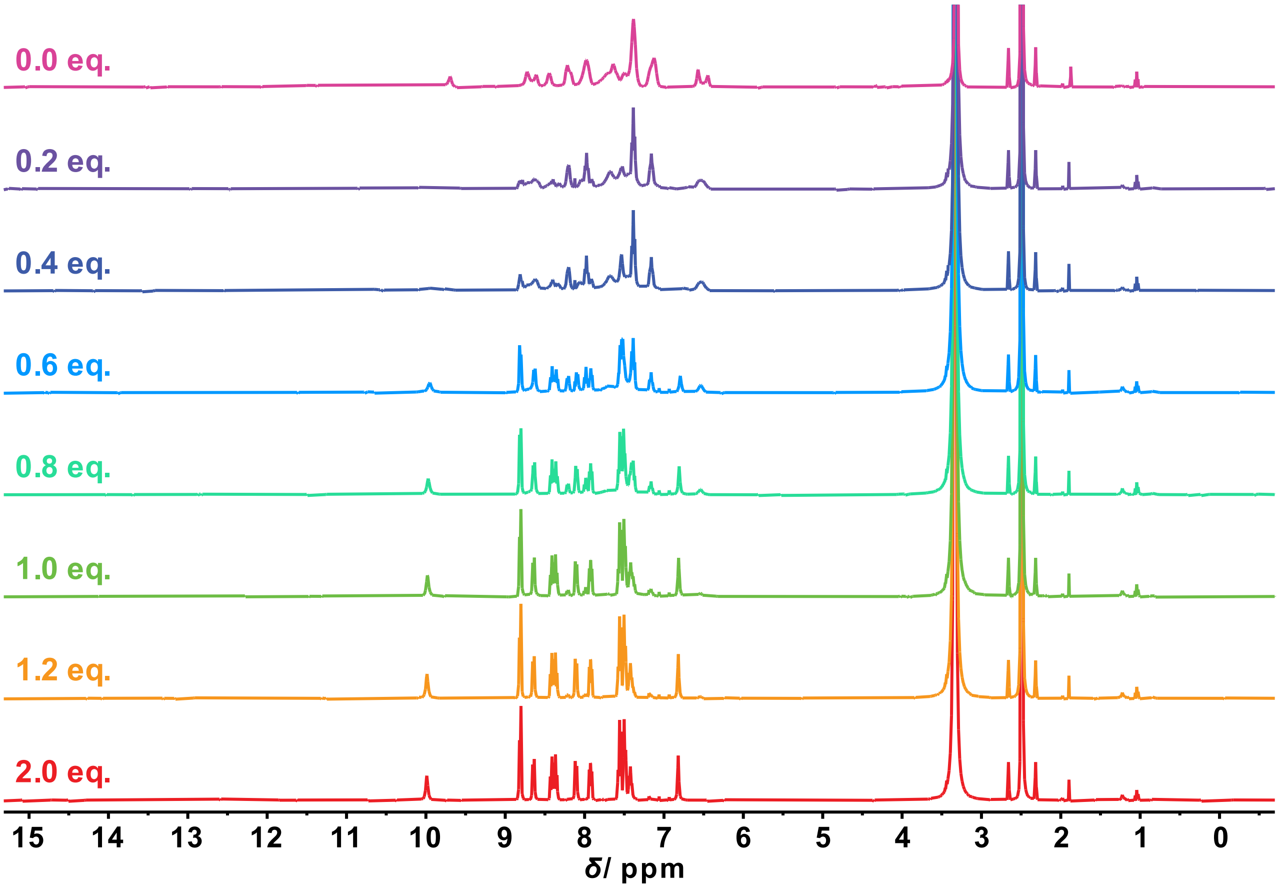

Fig. S10. Zoomed out NMR spectra of solutions of GFZnP BIPY in DMSO-*d6* containing different amounts of Zn^{2+} recorded at 400 MHz.

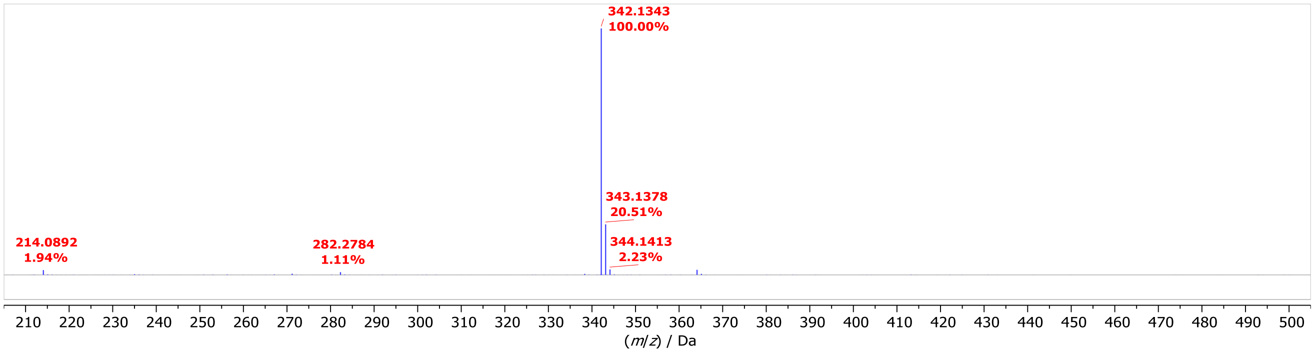

Fig. S11. HRMS spectrum of GFZnP BIPY.

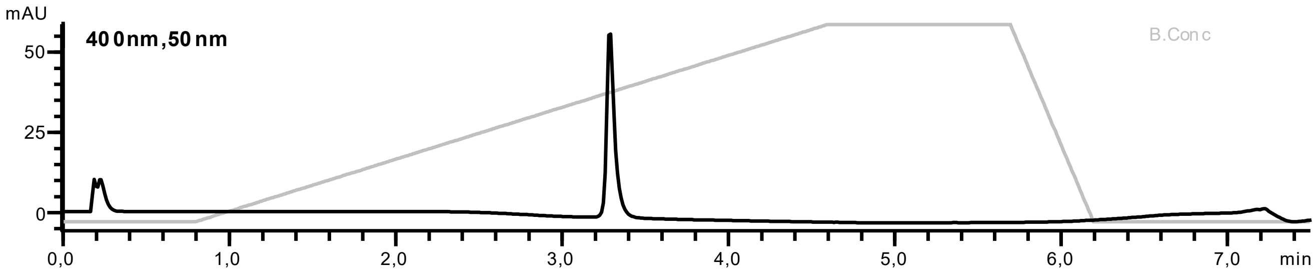

Fig. S12. HPLC-DAD chromatogram of GFZnP BIPY detected in the 350 - 450 nm absorption range, after 4 months of storage at room temperature in a 5 mM DMSO:EtOH 1:1 solution.

2. Additional spectroscopic data

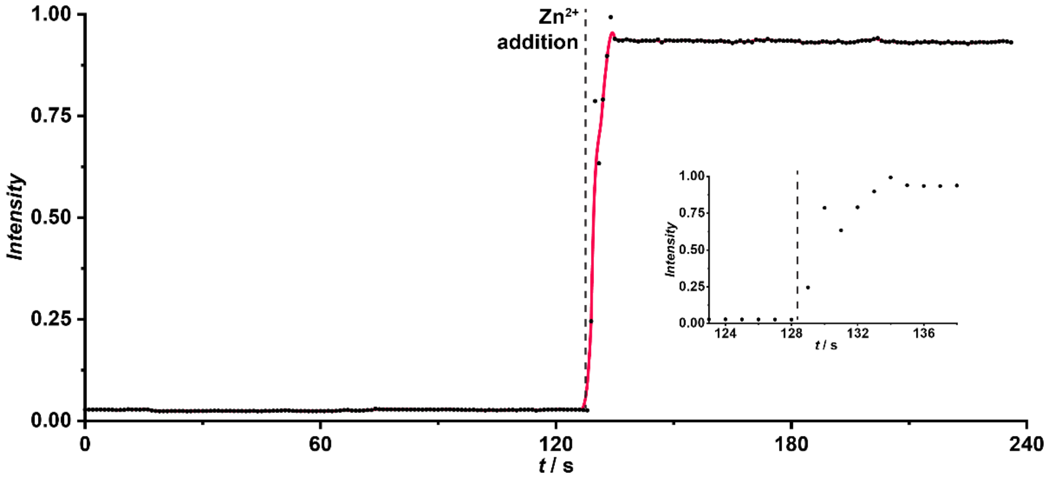

Fig. S13. Binding kinetics of the reported probe represented by a 4-minute time course measurement of 4 μM GFZnP BIPY in HEPES pH 7.4 buffer to which concentrated $Zn(OTf)_2$ solution was added (100x dilution from a 100 mM Zn^{2+} solution in deionized water, resulting in a 1 mM final concentration).

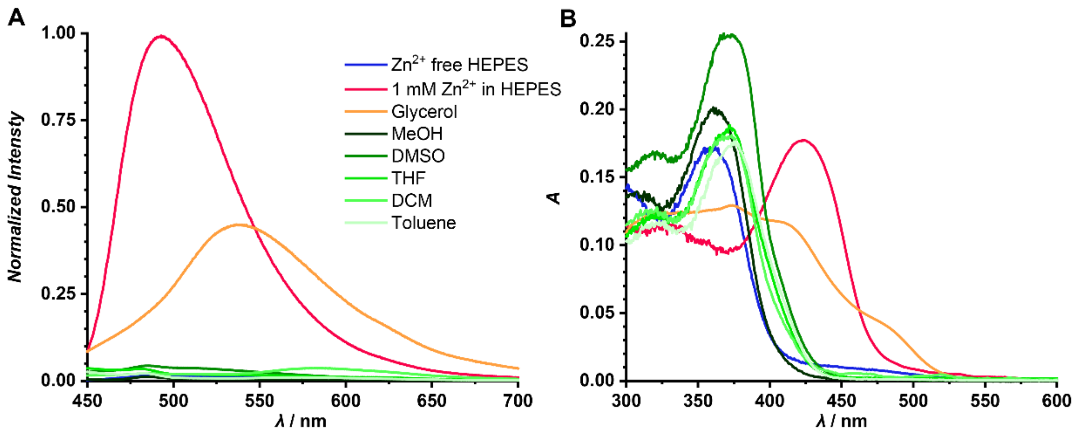

Fig. S14. (A) normalized fluorescence spcetra and (B) absorption spectra of 4 μM GFZnP BIPY in different solvents.

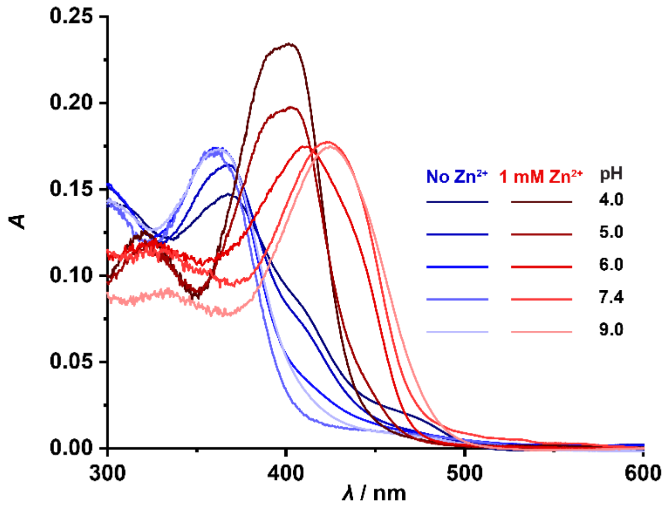

Fig. S15. Absorption spectra of 4 μM GFZnP BIPY in free (blue) and 1 mM Zn^{2+} containing (red) pH buffers.

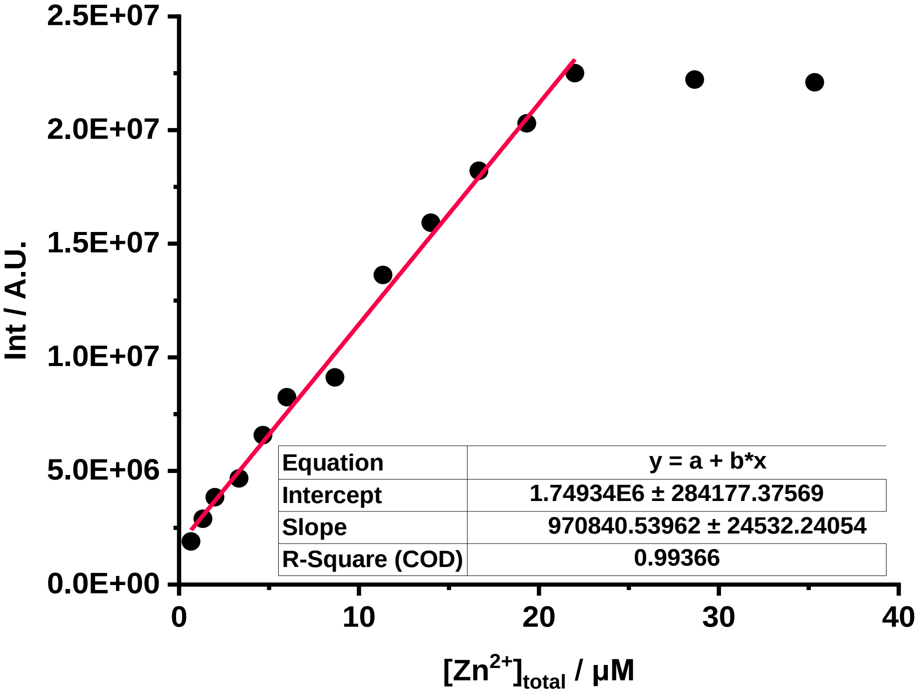

Fig. S16. Calibration line obtained by recording the fluorescence of GFZnP BIPY (33.3 µM in HEPES pH 7.4) in the presence of different quantities of total Zn^{2+} and the parameters of the fitted calibration line.

$$F_0 = 1894834,\ \sigma = 8933,\ s = 970840$$

$$LOD = (F_0 + 3\sigma / s) = 1.98\ \mu M = 129\ \mu g / L$$

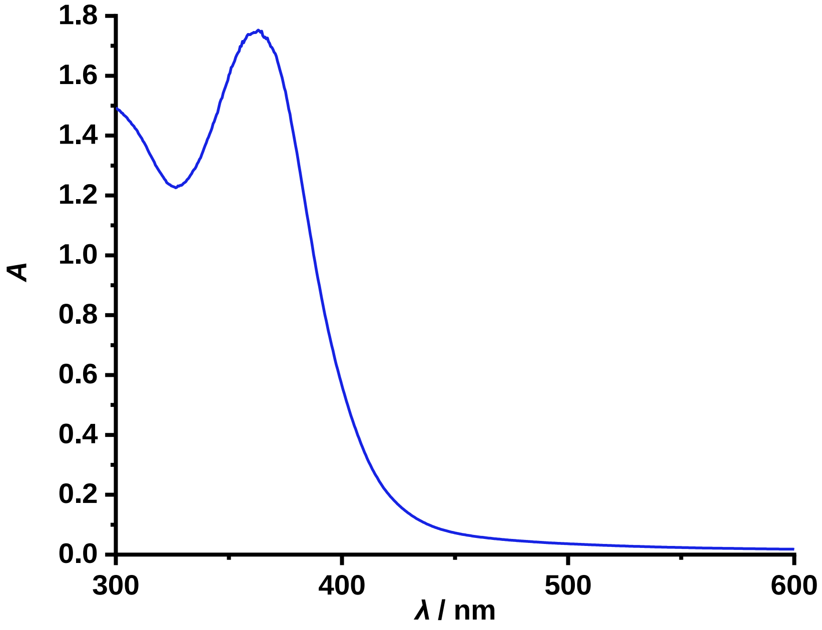

Fig. S17. Absorption spectrum of an aqueous saturated solution of GFZnP BIPY used for the determination of solubility.

3. Additional biological data

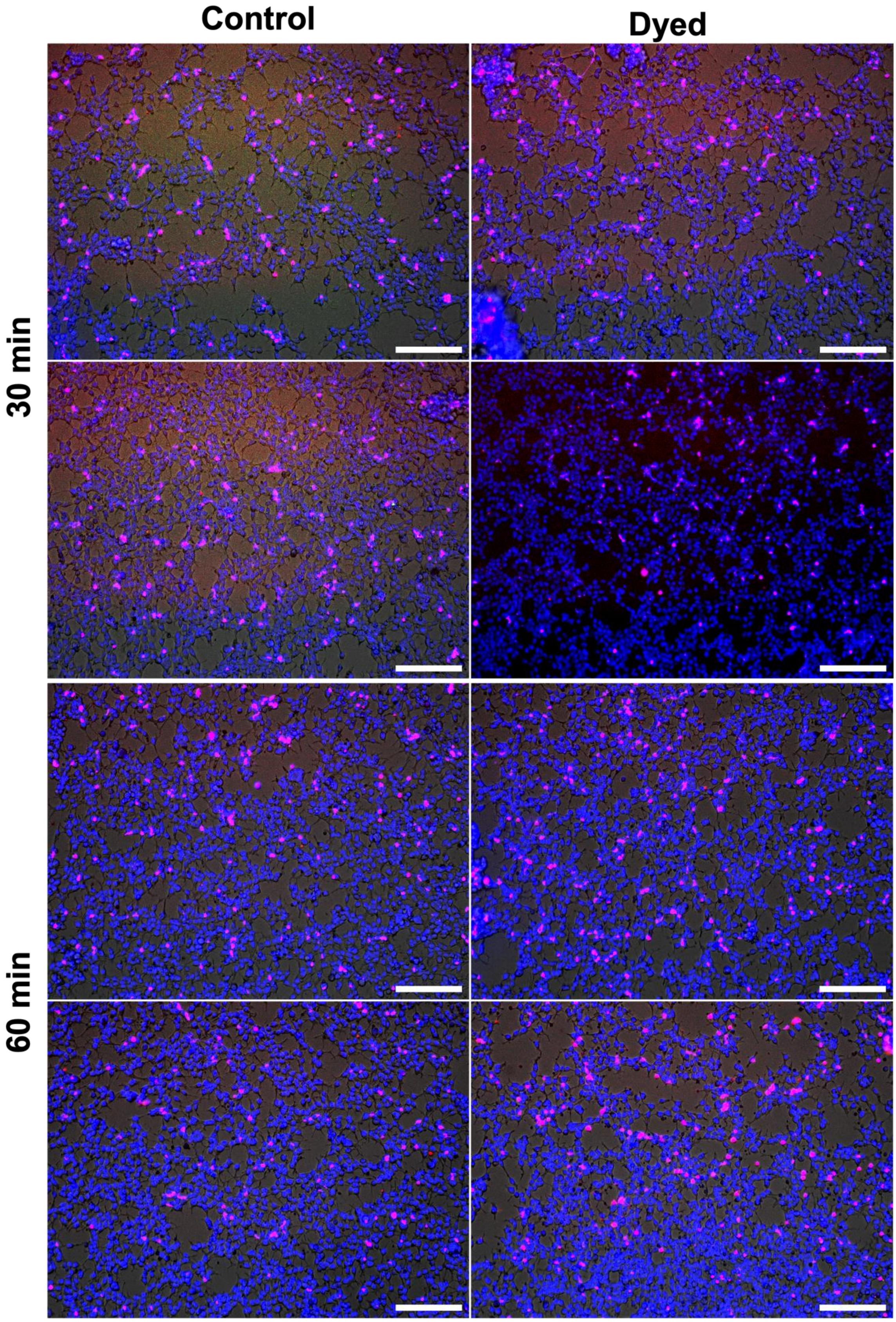

Fig. S18. Composite images of control and GFZnP BIPY dyed HEK293 cells 30 and 60 minutes after staining. Blue channel: Hoechst 33342, red channel: propidium iodide. Scale bars represent 200 μm.

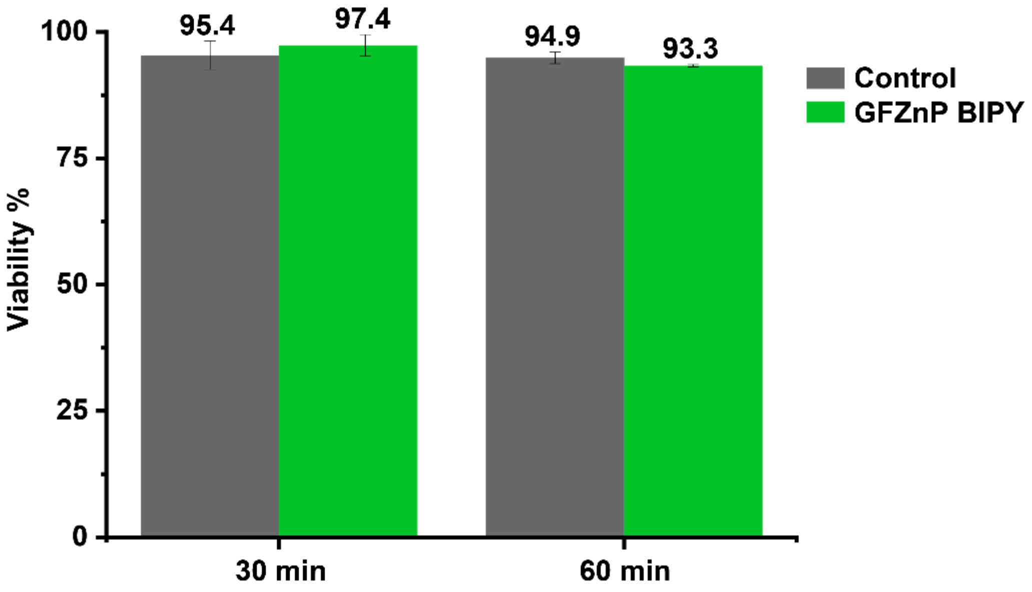

Fig. S19. Cell viabilities of HEK 293 cells stained with GFZnP BIPY measured 30 and 60 minutes after the staining. For both measurement lengths, one sample 10x magnification image was taken from two unstained control wells and two stained wells of a 24-well plate. Error bars represent the standard deviation. Each field of view contained 1441 – 2568 cells.

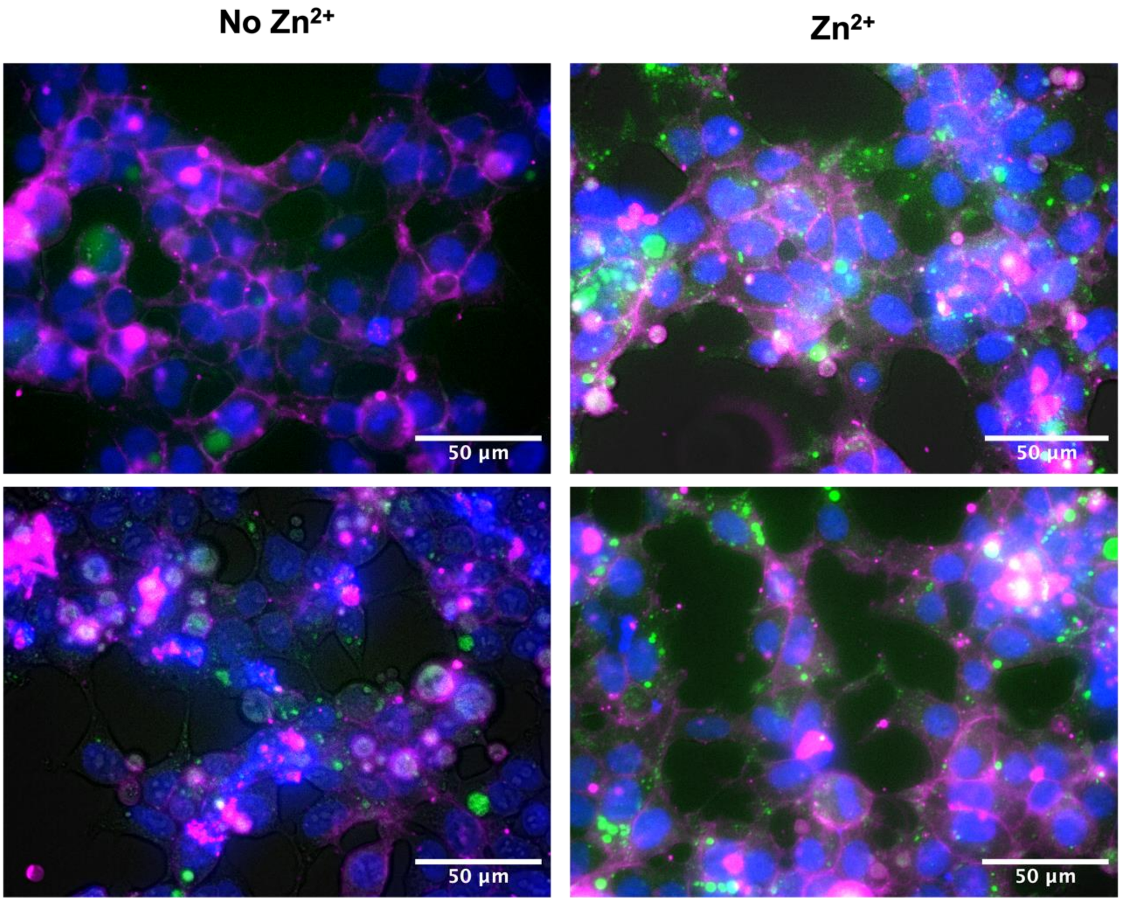

Fig. S20. Colocalization study of GFZnP BIPY (green), with the nuclei (blue) and cell membranes (purple).

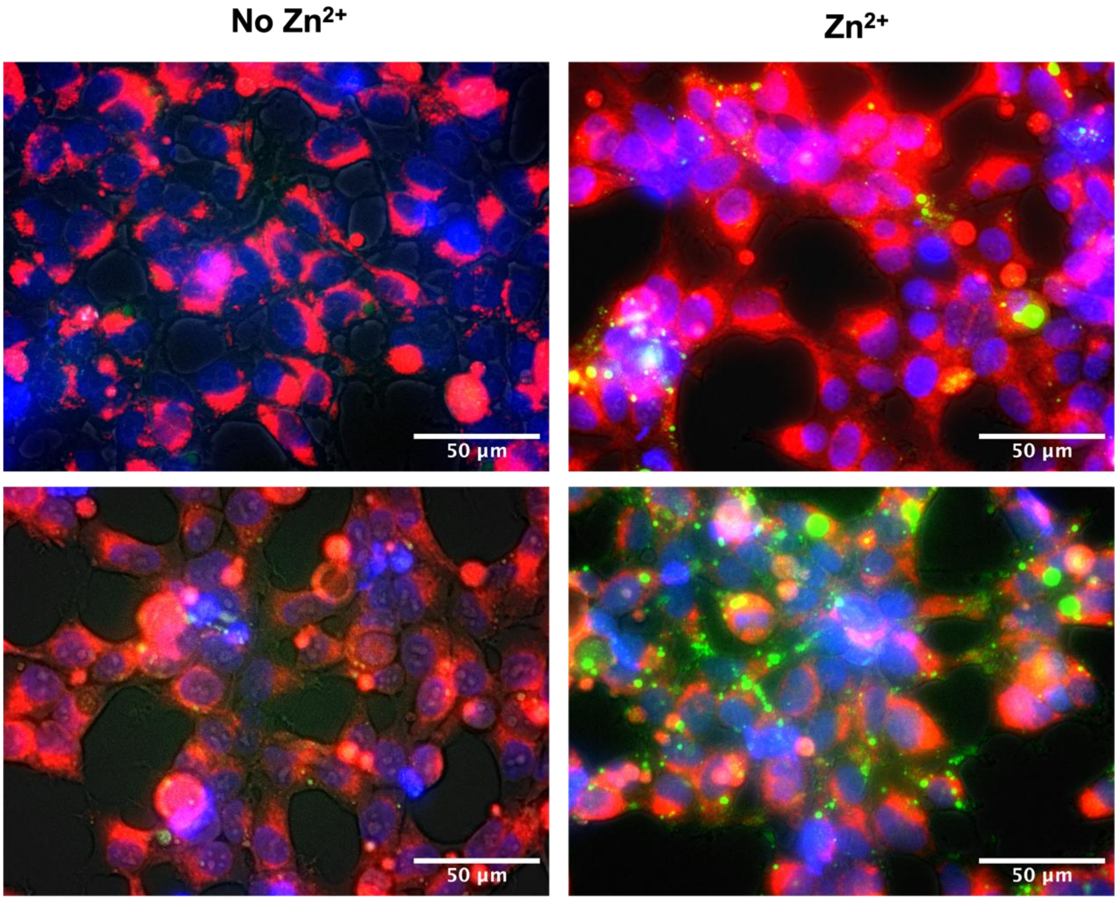

Fig. S21.Colocalization study of GFZnP BIPY (green), with the nuclei (blue) and mitochondria (red).

4. Theoretical studies

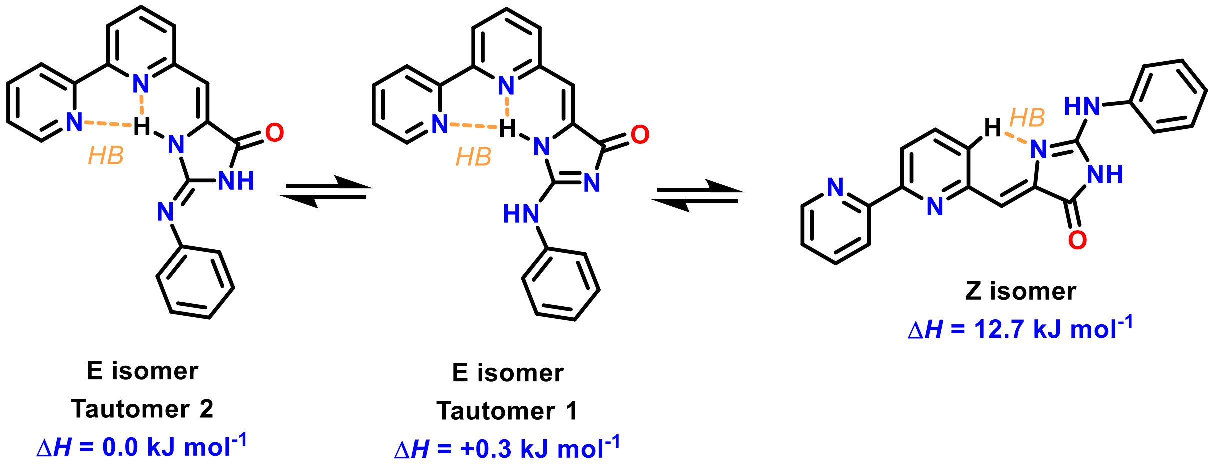

Scheme S1. Δ*H* difference between different *E* and *Z* geometries in the case **GFZnP BIPY**. Yellow lines show hydrogen bonds (HB) with stabilizing effects, that determine the most stable probe geometry. Calculation was carried out at M06-2X/6-311++G(2d,2p)//PCM(water) level of theory.

To model the complex formation, DFT calculations were used. The stability of each possible complex was estimated by the enthalpy of its formation from the most stable conformer of the free probe and a hexahydrate of Zn^{2+} as shown below:

Only complexation (Complex A, B, D): $\mathbf{Zn^{2+}(H_2O)_6 + L{-}H \rightarrow [L{-}H{-}Zn]^{2+} + 6\ H_2O}$

$\mathbf{\Delta H_c = (H_{[L-H-Zn]2+} + 6H_{H2O}) - (H_{[L-H]} + H_{Zn2+(H2O)6})}$

Complexation + deprotonation (Complex C, E, F):

$\mathbf{Zn^{2+}(H_2O)_6 + L{-}H \rightarrow [L{-}Zn]^{2+}\cdot x\ H_2O + H_3O^+ + 5{-}x\ H_2O}$

$\mathbf{\Delta H_c = [H_{[L-Zn]2+\cdot x\ H2O} + (5-x)H_{H2O} + H_{H3O+}] - (H_{[L-H]} + H_{Zn2+(H2O)6})}$

The free probe and complex were calculated at M06-2X/6-311++G(2d,2p)//PCM(water) level of theory.In these models, we needed to accurately predict the enthalpy of protonation (H_{H3O+}) and desolvatation (H_{H2O}) accurately. To earn this, modelling water and oxonium ions alone would not be enough, since in a condensed face both create stable hydrogen bonds with surrounding molecules, that need to be considered during the model creation. An accurate way to predict these quantities is to consider a multitude of water molecules from which a single water molecule is removed (H_{H2O}) or to which a proton is added (H_{H3O+}). The formation enthalpy of water was modelled using an 8-membered water cluster from which a single water molecule is removed as shown in **Scheme S2**.

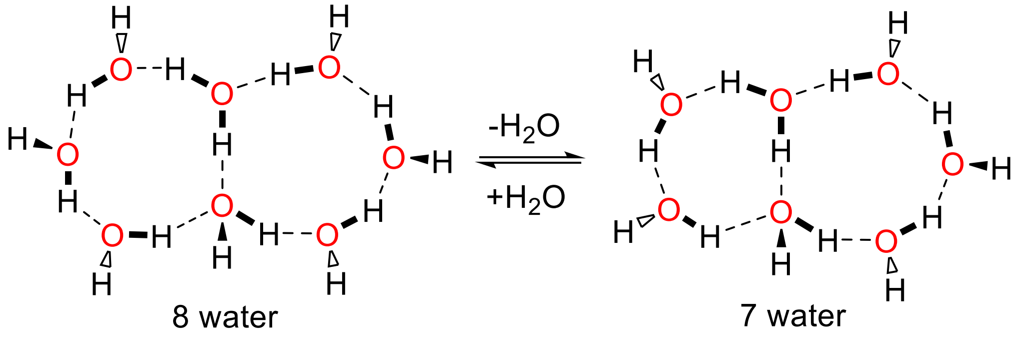

Scheme S2. The 8-membered waters clusters used for modelling solvation.

Table S1. Computed thermodynamic quantities of the 8-membered water clusters used for modelling solvation. ZPE is the zerus point energy, calculated at M06-2X/6-311++G(2d,2p) level of theory.

Quantity	ΔE	ΔZPE	ΔU	ΔH	ΔG	ΔS
kJ mol^{-1}	-200503.7	-200438.6	-200430.9	-200430.9	-200452.4	71.96
Hartree	-76.44060235	-76.415791	-76.412864	-76.412863	-76.421042	71.96

Analogously, a proton was added to the same 8-membered cluster to model the effect of deprotonation as shown in Scheme S3.

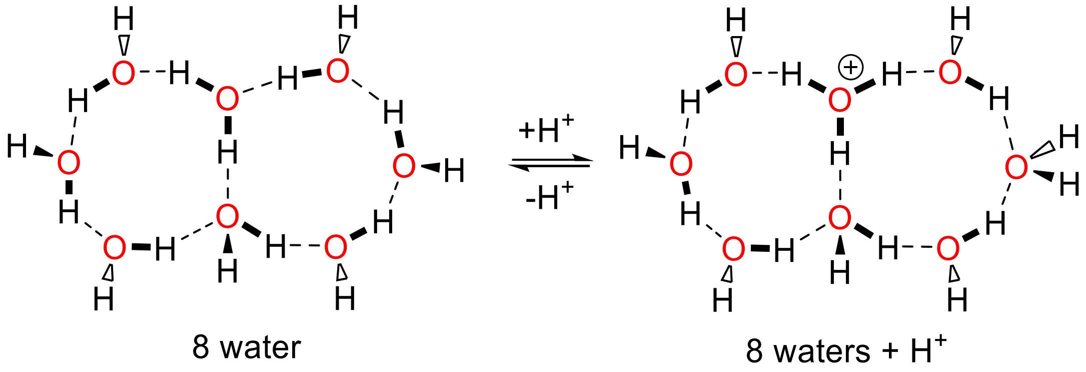

Scheme S3. The 8-membered waters clusters used for the proton solvation.

Table S2. Computed thermodynamic quantities of the 8-membered water clusters used for modelling the protonation. ZPE is the zerus point energy, calculated at M06-2X/6-311++G(2d,2p) level of theory.

Quantity	ΔE	ΔZPE	ΔU	ΔH	ΔG	ΔS
kJ mol^{-1}	-1131.9	-1100.9	-1103.5	-1103.5	-1095.6	-26.2
Hartree	-0.43152309	-0.419694	-0.420683	-0.420684	-0.417706	-26.2

The calculated Δ*H* values and the optimized probe and complex geometries are summarized in **Table S3.**

The protonation of heteroatoms in side-chains may further complicate the binding calculations, however, in this study we have not considered such effects.

Table S3. The sum enthalpy values relative to the free-form (ΔH_c) in kJ mol^{-1} for various complex models of selected probes calculated at M06-2X/6-311++G(2d,2p)//PCM(water) level of theory. λ_{abs} and λ_{em} , ε and *osc. str.* values were calculated at B3LYP/6-311++G(2d,2p)//PCM(water) level of theory, where *osc. str.* = oscillatory strength. Green fill color highlights the most stable species.

	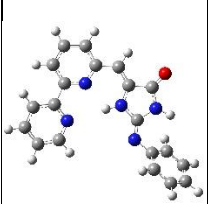	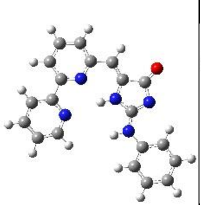	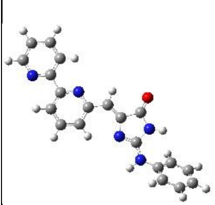	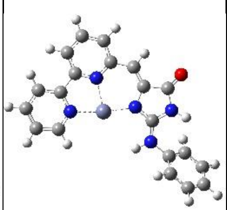	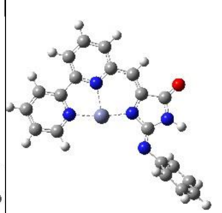	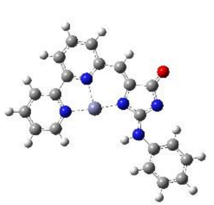	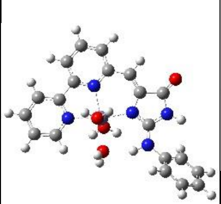	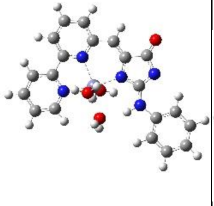	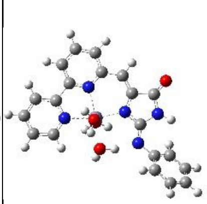
	**Free probe A L–H**	**Free probe B L–H In accordance with UV-VIS**	**Free probe C L–H**	**Complex A L–H : Zn^{2+}**	**Complex B L^- : Zn^{2+}**	**Complex C L^- : Zn^{2+}**	**Complex D L–H : Zn^{2+} : 3 H_2O**	**Complex E L^- : Zn^{2+} : 3 H_2O In accordance with NMR & UV-VIS**	**Complex F L^- : Zn^{2+} : 3 H_2O**
ΔH_c [kJ mol^{-1}]	0.0	0.3	12.7	-69.7	-40.4	-40.7	-121.5	**-82.9**	**-107.9**
λ_{abs} [nm] (*osc str.*)	Max 408<br>413 (0.918)	Max 394<br>403 (0.983)	423 (1.152)	419 (0.801)	max 438<br>477 (0.403)<br>424 (0.656)	max 432<br>461 (0.526)<br>405 (0.637)	**max 433**<br>468 (0.577)<br>413 (0.567)	**max 420**<br>**425 (0.836)**	**max 441**<br>**469 (0.637)**<br>**418 (0.487)**
ε [dm^3 mol^{-1} cm^{-1}]	38950	43200	47000	36000	37850	40700	39400	**36950**	**38900**
λ_{em} [nm] (*osc str.*)	440 (0.769)	472 (0.993)	516 (1.18)	469 (0.162)	max 519 (0.237)	max 432 (0.257)	454 (0.729)	**524 (0.976)**	**586 (0.900)**<br>**470 (0.201)**

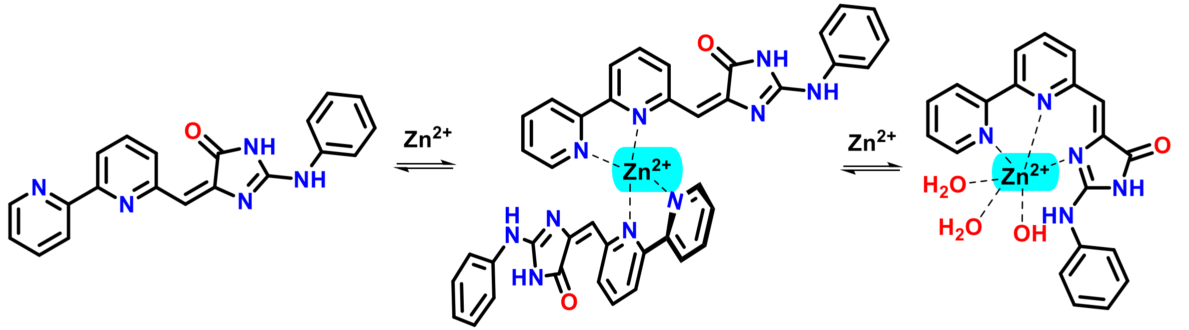

free

2 :1 complex
0.4-0.6 eq. Zn^{2+}

1 : 1 complex
>1 eq. Zn^{2+}

Fig. S22. Hypothesized complex equilibrium and complex structures of GFZnP BIPY at different Zn^{2+} equivalency (0.4-0.6 eq. and >1. eq.).

4.1 Raw computational data

Table S4. Computed energies (*E*), zero point energies, internal energies (*U*), enthalpies (*H*) and Gibbs free energies (*G*) given in Hartree as well as entropies (*S*) given in J mol^{-1} K^{-1} at M06-2X/6-311++G(2d,2p) basis set with the consideration of PCM solvent method using the parameter set of water for **small molecules and ions**.

Name	Filename	E	ZPE	U	H	G	S
7 H_2O	000aaa_5_H2O_+2H2O_M062X_6311++2d2p_PCMw.log	-535.08135498	-534.908272	-534.889627	-534.888683	-534.956561	142.860
8 H_2O	000aaa_5_H2O_+3H2O_M062X_6311++2d2p_PCMw.log	-611.52195733	-611.324063	-611.302491	-611.301546	-611.377603	160.075
8 H_2O +H^+	001aaa_5_H2O_+3H2O_+H+_M062X_6311++2d2p_PCMw.log	-611.95348042	-611.743757	-611.723174	-611.722230	-611.795309	153.808
$Zn^{2+}(H_2O)_6$	000aaa_Zn2+_6H2O_M062X_6311++2d2p_PCMw.log	-2237.69628717	-2237.546664	-2237.528735	-2237.527791	-2237.590689	132.381

Table S5. Computed energies (*E*), zero point energies, internal energies (*U*), enthalpies (*H*) and Gibbs free energies (*G*) given in Hartree as well as entropies (*S*) given in J mol^{-1} K^{-1} at M06-2X/6-311++G(2d,2p) basis set with the consideration of PCM solvent method using the parameter set of water for compound.

Number	Name	Filename	E	ZPE	U	H	G	S
**free probe A**	isomer-E, tautomer-2,	_801ace_GFP_bipiridil_M062X_6311++2d2p_PCMw.log	-1120.07413551	-1119.755437	-1119.735233	-1119.734289	-1119.807811	154.740
**free probe B**	isomer-E tautomer-1	_801abe_GFP_bipiridil_M062X_6311++2d2p_PCMw.log	-1120.07440272	-1119.755312	-1119.735110	-1119.734166	-1119.807820	155.017
**free probe C**	isomer-Z tautomer-1	_801aad_GFP_bipiridil_M06_2X_6311++2d2p_PCMw.log	-1120.06950057	-1119.750715	-1119.730266	-1119.729322	-1119.804671	158.585
**Complex A**		_803aae_GFP_bipiridil_Zn2+_M062X_6311++2d2p_PCMw.log	-2899.15712235	-2898.833503	-2898.812282	-2898.811338	-2898.885204	155.463
**Complex B**		_803bae_GFP_bipiridil-H+_Zn2+_M062X_6311++2d2p_PCMw.log	-2898.71237664	-2898.402631	-2898.381573	-2898.380629	-2898.454350	155.159
**Complex C**		_803bbe_GFP_bipiridil-H+_Zn2+_M062X_6311++2d2p_PCMw.log	-2898.71243843	-2898.401332	-2898.380561	-2898.379617	-2898.452427	153.241
**Complex D**		_804aae_GFP_bipiridil_Zn2+_+3H2O_M062X_6311++2d2p_PCMw.log	-3128.49908251	-3128.101036	-3128.070629	-3128.069685	-3128.162989	196.375
**Complex E**		_805aae_GFP_bipiridil-1H+_Zn2+_+3H2O_M062X_6311++2d2p_PCMw.log	-3128.05083664	-3127.665069	-3127.635221	-3127.634277	-3127.725978	193.001
**Complex F**		_805abe_GFP_bipiridil-1H+_Zn2+_+3H2O_M062X_6311++2d2p_PCMw.log	-3128.05757617	-3127.673159	-3127.643487	-3127.642543	-3127.734174	192.854

4.2 The coordinates of computed geometries

7 membered water cluster : 000aaa_5_H2O_+2H2O_M062X_6311++2d2p_PCMw.log

orientation:

Center Number	Atomic Number	Atomic Type	Coordinates (Angstroms) X	Y	Z
1	8	0	-1.587073	-2.313966	-0.024587
2	1	0	-0.743333	-1.972492	0.344442
3	8	0	0.671050	-1.287793	1.087300
4	1	0	0.557219	-0.324001	1.126061
5	8	0	0.432855	1.532116	0.965444
6	1	0	-0.422202	1.787392	0.549524
7	8	0	-1.928651	2.121810	-0.183414
8	1	0	-2.427043	1.292158	-0.339418
9	8	0	-3.261032	-0.224602	-0.567438
10	1	0	-2.682621	-0.990125	-0.368506
11	8	0	2.642949	1.376802	-0.722218
12	1	0	0.505142	2.037463	1.779151
13	1	0	3.358447	1.937413	-0.412756
14	1	0	-1.349704	-2.807774	-0.812936
15	1	0	-4.038046	-0.329436	-0.013467
16	1	0	1.472496	-1.421656	0.551173
17	1	0	1.893950	1.543483	-0.121891
18	1	0	-1.859609	2.555000	-1.037517
19	8	0	3.008137	-1.347256	-0.465027
20	1	0	2.974528	-0.388022	-0.643557
21	1	0	2.934897	-1.776275	-1.320776

8 membered water cluster : 000aaa_5_H2O_+3H2O_M062X_6311++2d2p_PCMw.log

orientation:

Center Number	Atomic Number	Atomic Type	Coordinates (Angstroms) X	Y	Z
1	8	0	-2.197939	-2.273595	-0.196220
2	1	0	-1.374683	-1.973630	0.249636
3	8	0	-0.000568	-1.385547	1.116234
4	1	0	0.003020	-0.414690	1.118355
5	8	0	-0.081445	1.451678	1.087843
6	1	0	-0.921439	1.723197	0.650818
7	8	0	-2.387585	2.134343	-0.122215
8	1	0	-2.907536	1.337812	-0.360249
9	8	0	-3.783679	-0.121740	-0.735667
10	1	0	-3.239460	-0.913022	-0.539159
11	8	0	2.220624	2.224103	-0.272164
12	1	0	-0.105121	1.828409	1.971705
13	1	0	1.965157	2.699795	-1.066108
14	1	0	-1.915740	-2.727879	-0.993489
15	1	0	-4.594476	-0.226552	-0.232409
16	1	0	0.843220	-1.659891	0.712752
17	1	0	1.388284	1.989021	0.178075
18	1	0	-2.272339	2.629883	-0.936484
19	8	0	2.412464	-2.208447	-0.008087
20	1	0	2.935644	-1.429045	-0.282308
21	1	0	2.293055	-2.734147	-0.802450
22	8	0	3.818804	0.038022	-0.729150
23	1	0	4.626694	0.169379	-0.227432
24	1	0	3.270315	0.830827	-0.565848

hydrated Zn ion: 000aaa_Zn2+_6H2O_M062X_6311++2d2p_PCMw.log

orientation:

Center Number	Atomic Number	Atomic Type	Coordinates (Angstroms) X	Y	Z
1	30	0	-0.000716	-0.001877	-0.006270
2	8	0	1.306408	1.088909	1.235839
3	1	0	1.898598	0.523166	1.744832
4	8	0	-1.294355	1.643087	-0.243744
5	1	0	-1.913382	1.534864	-0.975361
6	8	0	1.337334	0.504679	-1.550789
7	1	0	1.023236	0.693619	-2.441662
8	8	0	-1.323860	-1.015432	-1.291306
9	1	0	-1.923157	-1.605997	-0.820293
10	8	0	1.302430	-1.619231	0.347487
11	1	0	0.974985	-2.483571	0.618485
12	8	0	-1.325817	-0.595382	1.521155
13	1	0	-1.915518	0.113031	1.804966
14	1	0	-1.006579	-1.025375	2.321940
15	1	0	-1.013008	-1.497691	-2.065055
16	1	0	-0.948699	2.540485	-0.304729
17	1	0	1.933270	1.223455	-1.309233
18	1	0	1.919209	-1.772181	-0.377885
19	1	0	0.975383	1.759449	1.842956

8 membered protonated water cluster : 001aab_5_H2O_+3H2O_+H+_M062X_6311++2d2p_PCMw.log

orientation:

Center Number	Atomic Number	Atomic Type	Coordinates (Angstroms) X	Y	Z
1	8	0	-2.108543	-2.085739	0.069068
2	1	0	-0.846857	-1.606691	0.726539
3	8	0	0.022571	-1.299415	1.172632
4	1	0	0.038047	-0.290542	1.233144
5	8	0	0.067339	1.269756	1.307429
6	1	0	-0.706480	1.650172	0.816052
7	8	0	-2.004820	2.226238	-0.032309
8	1	0	-2.516769	1.522799	-0.464900
9	8	0	-3.455881	0.048566	-1.114420
10	1	0	-2.629331	-1.368271	-0.337794
11	8	0	2.496691	2.045948	0.049794
12	1	0	0.005283	1.590294	2.212837
13	1	0	2.345513	2.832294	-0.480637
14	1	0	-2.699718	-2.546110	0.671788
15	1	0	-4.389140	0.100576	-0.882832
16	1	0	0.858143	-1.650990	0.646368
17	1	0	1.651465	1.855516	0.488405
18	1	0	-1.780877	2.856665	-0.721987
19	8	0	1.986324	-2.164382	-0.068839
20	1	0	2.494881	-1.449409	-0.533165
21	1	0	2.605378	-2.615111	0.512679
22	8	0	3.222077	-0.197195	-1.331928
23	1	0	4.180098	-0.262305	-1.355295
24	1	0	3.015687	0.639872	-0.867253
25	1	0	-3.431401	-0.008983	-2.075370

free probe C: _801aad_GFP_bipiridil_M06_2X_6311++2d2p_PCMw.log

Standard orientation:

Center Number	Atomic Number	Atomic Type	Coordinates (Angstroms) X	Y	Z
1	6	0	3.685183	-2.051734	0.178691
2	6	0	2.385436	-2.528721	0.224275
3	6	0	1.327735	-1.638422	0.150185
4	6	0	1.611477	-0.272986	0.030691
5	6	0	3.882942	-0.676085	0.058901
6	6	0	0.580685	0.756972	-0.057215
7	6	0	-0.753952	0.608624	-0.047470
8	1	0	0.945894	1.772249	-0.143659
9	7	0	-1.534015	-0.537696	0.050511
10	6	0	-1.656785	1.803569	-0.159833
11	8	0	-1.396466	2.976019	-0.285443
12	6	0	-2.773576	-0.127162	0.009163
13	7	0	-2.920470	1.243655	-0.091655
14	7	0	-3.831903	-0.952683	0.044110
15	1	0	-3.603601	-1.932036	-0.044811
16	6	0	-5.198936	-0.578017	0.041969
17	6	0	-6.075638	-1.240733	-0.812212
18	6	0	-5.671911	0.405820	0.905978
19	6	0	-7.421972	-0.908084	-0.808741
20	1	0	-5.697724	-2.007310	-1.474512
21	6	0	-7.017192	0.750108	0.882648
22	1	0	-4.999202	0.880333	1.606963
23	6	0	-7.895435	0.095371	0.029085
24	1	0	-8.099805	-1.427149	-1.471353
25	1	0	-7.379117	1.518993	1.550213
26	1	0	-8.943143	0.359142	0.021901
27	1	0	-3.783904	1.745378	-0.226825
28	1	0	4.535538	-2.712118	0.232712
29	1	0	2.197400	-3.589344	0.316926
30	1	0	0.303465	-1.972382	0.181932
31	7	0	2.871612	0.187478	-0.012868
32	6	0	5.262241	-0.105717	0.004658
33	6	0	5.462105	1.268651	-0.116382
34	6	0	6.758760	1.753580	-0.164761
35	1	0	4.610156	1.927478	-0.169977
36	6	0	7.518472	-0.493877	0.029429
37	6	0	7.814672	0.857343	-0.090906
38	1	0	6.941806	2.814853	-0.258716
39	1	0	8.315620	-1.224542	0.090542
40	1	0	8.841749	1.188851	-0.124848
41	7	0	6.277740	-0.972403	0.076778

free probe B: _801abe_GFP_bipiridil_M062X_6311++2d2p_PCMw.log

Standard orientation:

Center Number	Atomic Number	Atomic Type	Coordinates (Angstroms) X	Y	Z
1	6	0	4.705349	-0.455827	-0.131484
2	6	0	4.867843	-1.834809	-0.150587
3	6	0	3.758013	-2.660257	-0.082827
4	6	0	2.497375	-2.064161	-0.001808
5	6	0	3.412654	0.059121	-0.034650
6	6	0	1.263891	-2.843681	0.044679
7	6	0	0.045184	-2.289383	0.047056
8	1	0	1.316714	-3.923215	0.066460
9	7	0	-0.317662	-0.957013	0.008258
10	6	0	-1.275516	-3.019132	0.077948
11	8	0	-1.409255	-4.226720	0.117369
12	6	0	-1.684063	-0.904722	0.011264
13	7	0	-2.286470	-2.081569	0.052149
14	7	0	-2.275930	0.295835	-0.027322
15	1	0	-1.645241	1.083649	-0.062833
16	6	0	-3.647457	0.628612	-0.025105
17	6	0	-3.953424	1.990432	-0.065901
18	6	0	-4.674234	-0.312614	0.015182
19	6	0	-5.273186	2.408656	-0.066254
20	1	0	-3.153146	2.718557	-0.096947
21	6	0	-5.993731	0.124628	0.014412
22	1	0	-4.443055	-1.362904	0.046304
23	6	0	-6.304130	1.476575	-0.025771
24	1	0	-5.494038	3.466137	-0.097987
25	1	0	-6.785806	-0.610502	0.045958
26	1	0	-7.334423	1.801604	-0.025766
27	1	0	5.566308	0.189972	-0.201620
28	1	0	5.858622	-2.260129	-0.224190
29	1	0	3.854256	-3.736086	-0.100427
30	7	0	2.352489	-0.736536	0.026900
31	6	0	3.108393	1.522439	0.000567
32	6	0	4.108524	2.475886	0.175152
33	6	0	3.762189	3.817985	0.203307
34	1	0	5.139163	2.182231	0.300856
35	6	0	1.499724	3.150363	-0.105812
36	6	0	2.430255	4.169099	0.057643
37	1	0	4.522274	4.573813	0.340814
38	1	0	0.448594	3.385909	-0.220100
39	1	0	2.111953	5.200626	0.072863
40	7	0	1.818285	1.859545	-0.133275
41	1	0	0.354968	-0.192997	-0.011382

free probe A: _801ace_GFP_bipiridil_M062X_6311++2d2p_PCMw.log

Standard orientation:

Center Number	Atomic Number	Atomic Type	Coordinates (Angstroms) X	Y	Z
1	6	0	-4.687787	-0.768410	0.148912
2	6	0	-4.705385	-2.157216	0.129451
3	6	0	-3.516327	-2.857451	0.025455
4	6	0	-2.325140	-2.129706	-0.050172
5	6	0	-3.458011	-0.118004	0.053106
6	6	0	-1.023658	-2.779951	-0.129979
7	6	0	0.137204	-2.107864	-0.123009
8	1	0	-0.969246	-3.857775	-0.184023
9	7	0	0.385482	-0.754238	-0.046106
10	6	0	1.482660	-2.759792	-0.193096
11	8	0	1.742841	-3.935623	-0.283432
12	6	0	1.732824	-0.480865	-0.053020
13	7	0	2.376402	-1.717685	-0.133365
14	7	0	2.226979	0.687999	-0.020607
15	6	0	3.626009	0.853709	0.022340
16	6	0	4.233332	1.680551	-0.924777
17	6	0	4.412520	0.276230	1.023122
18	6	0	5.602578	1.899404	-0.888724
19	1	0	3.618717	2.142105	-1.685217
20	6	0	5.782713	0.503586	1.056043
21	1	0	3.942660	-0.332478	1.784809
22	6	0	6.385097	1.310843	0.099415
23	1	0	6.060050	2.535688	-1.633754
24	1	0	6.377858	0.051938	1.837706
25	1	0	7.450842	1.487089	0.128058
26	1	0	-5.609282	-0.216942	0.251084
27	1	0	-5.644846	-2.686519	0.202497
28	1	0	-3.497778	-3.937552	0.011508
29	7	0	-2.318085	-0.793187	-0.041423
30	6	0	-3.318393	1.370044	0.056224
31	6	0	-4.412347	2.208483	-0.145724
32	6	0	-4.218875	3.581237	-0.136566
33	1	0	-5.396153	1.802359	-0.323320
34	6	0	-1.909582	3.164767	0.257189
35	6	0	-2.941928	4.076163	0.072011
36	1	0	-5.052883	4.249979	-0.295340
37	1	0	-0.896721	3.512939	0.418937
38	1	0	-2.741817	5.137017	0.087756
39	7	0	-2.081633	1.846262	0.249201
40	1	0	-0.350561	-0.056721	0.008431
41	1	0	3.377380	-1.824745	-0.184786

Complex A: _803aae_GFP_bipiridil_Zn2+_M062X_6311++2d2p_PCMw.log

Standard orientation:

Center Number	Atomic Number	Atomic Type	Coordinates (Angstroms) X	Y	Z
1	6	0	4.588515	1.334359	-0.040113
2	6	0	4.344948	2.699331	-0.079353
3	6	0	3.042186	3.156639	-0.084821
4	6	0	1.996345	2.231893	-0.053316
5	6	0	3.506510	0.465696	-0.008313
6	6	0	0.624351	2.712761	-0.059019
7	6	0	-0.508447	2.000349	-0.061188
8	1	0	0.492407	3.786477	-0.062154
9	7	0	-0.732309	0.624176	-0.061672
10	6	0	-1.843490	2.679147	-0.063276
11	8	0	-2.112611	3.847973	-0.078228
12	6	0	-2.053092	0.442787	-0.064641
13	7	0	-2.741951	1.618985	-0.048100
14	7	0	-2.657283	-0.732039	-0.089878
15	1	0	-2.074491	-1.546109	-0.221429
16	6	0	-4.074448	-0.920774	-0.023946
17	6	0	-4.704999	-1.629431	-1.037966
18	6	0	-4.790354	-0.421300	1.056968
19	6	0	-6.075512	-1.833775	-0.968447
20	1	0	-4.126259	-2.009613	-1.867881
21	6	0	-6.164736	-0.613205	1.105159
22	1	0	-4.276243	0.096997	1.855025
23	6	0	-6.806721	-1.320184	0.096487
24	1	0	-6.572535	-2.386475	-1.752456
25	1	0	-6.727831	-0.222586	1.940285
26	1	0	-7.875026	-1.475122	0.142289
27	1	0	-3.746204	1.708116	-0.098903
28	1	0	5.600985	0.966837	-0.034785
29	1	0	5.169687	3.396177	-0.104755
30	1	0	2.818635	4.212291	-0.114116
31	7	0	2.245679	0.913956	-0.015582
32	6	0	3.672691	-1.014633	0.037216
33	6	0	4.914362	-1.633094	0.052104
34	6	0	4.981389	-3.017203	0.096889
35	1	0	5.821652	-1.052736	0.029657
36	6	0	2.606028	-3.077115	0.108023
37	6	0	3.810424	-3.756375	0.125947
38	1	0	5.942860	-3.509243	0.108973
39	1	0	1.662712	-3.601908	0.128683
40	1	0	3.819815	-4.834338	0.161429
41	7	0	2.545368	-1.743575	0.064619
42	30	0	0.798617	-0.666610	0.025463

Complex B: _803bae_GFP_bipiridil-H+_Zn2+_M062X_6311++2d2p_PCMw.log

Standard orientation:

Center Number	Atomic Number	Atomic Type	Coordinates (Angstroms) X	Y	Z
1	6	0	4.569764	1.308309	-0.001616
2	6	0	4.334211	2.681159	-0.033442
3	6	0	3.041469	3.149907	-0.058124
4	6	0	1.973216	2.236908	-0.049415
5	6	0	3.480892	0.456178	0.004759
6	6	0	0.614291	2.726607	-0.075816
7	6	0	-0.520026	1.993586	-0.072731
8	1	0	0.482739	3.798783	-0.101154
9	7	0	-0.724077	0.643811	-0.042776
10	6	0	-1.870165	2.661513	-0.109117
11	8	0	-2.126282	3.841587	-0.154876
12	6	0	-2.078514	0.390796	-0.049140
13	7	0	-2.752190	1.617556	-0.081685
14	7	0	-2.584656	-0.778055	-0.052506
15	6	0	-3.984436	-0.928641	-0.000831
16	6	0	-4.621657	-1.678614	-0.991683
17	6	0	-4.747439	-0.406082	1.048206
18	6	0	-5.994620	-1.873666	-0.950168
19	1	0	-4.027618	-2.097451	-1.792239
20	6	0	-6.121083	-0.609318	1.086389
21	1	0	-4.255249	0.144752	1.839290
22	6	0	-6.752309	-1.338651	0.086517
23	1	0	-6.474948	-2.447865	-1.730472
24	1	0	-6.696855	-0.199078	1.904636
25	1	0	-7.821079	-1.495056	0.118110
26	1	0	-3.754754	1.713458	-0.118894
27	1	0	5.579070	0.932839	0.018772
28	1	0	5.165957	3.370569	-0.038527
29	1	0	2.830628	4.208505	-0.083754
30	7	0	2.220203	0.915492	-0.017207
31	6	0	3.626304	-1.027044	0.036627
32	6	0	4.860091	-1.664360	0.037828
33	6	0	4.909292	-3.048710	0.066388
34	1	0	5.774553	-1.095144	0.014611
35	6	0	2.534232	-3.077769	0.090085
36	6	0	3.728273	-3.773514	0.093624
37	1	0	5.864135	-3.553822	0.066478
38	1	0	1.583515	-3.589606	0.109495
39	1	0	3.723575	-4.851907	0.115989
40	7	0	2.489804	-1.742384	0.062013
41	30	0	0.753409	-0.641272	0.029725

Complex C: _803bbe_GFP_bipiridil-H+_Zn2+_M062X_6311++2d2p_PCMw.log

Standard orientation:

Center Number	Atomic Number	Atomic Type	Coordinates (Angstroms) X	Y	Z
1	6	0	4.630231	1.132235	0.120570
2	6	0	4.470886	2.515980	0.096589
3	6	0	3.208830	3.061315	0.013808
4	6	0	2.094805	2.211154	-0.049024
5	6	0	3.497907	0.341401	0.046880
6	6	0	0.755854	2.763907	-0.097158
7	6	0	-0.418239	2.103591	-0.107726
8	1	0	0.679611	3.842568	-0.093390
9	7	0	-0.715592	0.761111	-0.107944
10	6	0	-1.757149	2.823683	-0.090692
11	8	0	-1.905234	4.031653	-0.101043
12	6	0	-2.087769	0.709624	-0.072148
13	7	0	-2.737469	1.868679	-0.059175
14	7	0	-2.692598	-0.487905	-0.055350
15	6	0	-4.075277	-0.775349	0.010675
16	6	0	-4.495276	-1.994221	-0.520773
17	6	0	-4.997134	0.070302	0.623614
18	6	0	-5.829640	-2.362316	-0.448858
19	1	0	-3.773637	-2.649325	-0.991054
20	6	0	-6.332383	-0.307543	0.681849
21	1	0	-4.671780	1.006096	1.045558
22	6	0	-6.757456	-1.517794	0.148916
23	1	0	-6.142553	-3.309345	-0.865240
24	1	0	-7.043481	0.352523	1.158702
25	1	0	-7.798476	-1.802106	0.202685
26	1	0	5.614509	0.702059	0.200604
27	1	0	5.338620	3.157275	0.149714
28	1	0	3.061032	4.130701	0.003187
29	7	0	2.273745	0.880603	-0.042287
30	6	0	3.543682	-1.150457	0.075689
31	6	0	4.733320	-1.863498	0.107766
32	6	0	4.692391	-3.248764	0.137070
33	1	0	5.682672	-1.353604	0.101935
34	6	0	2.318965	-3.126376	0.098432
35	6	0	3.467357	-3.896016	0.136528
36	1	0	5.612362	-3.814463	0.157694
37	1	0	1.338020	-3.577698	0.087794
38	1	0	3.393949	-4.971881	0.158165
39	7	0	2.360339	-1.792226	0.069579
40	30	0	0.754784	-0.541018	-0.148140
41	1	0	-2.094921	-1.292456	-0.169624

Complex D: _804aae_GFP_bipiridil_Zn2+_+3H2O_M062X_6311++2d2p_PCMw.log

Standard orientation:

Center Number	Atomic Number	Atomic Type	Coordinates (Angstroms) X	Y	Z
1	6	0	4.528149	1.604038	0.000518
2	6	0	4.243385	2.959366	-0.044087
3	6	0	2.926669	3.369474	-0.076287
4	6	0	1.910453	2.411078	-0.061406
5	6	0	3.476314	0.696832	0.010904
6	6	0	0.532453	2.876961	-0.094314
7	6	0	-0.605199	2.171216	-0.079554
8	1	0	0.401522	3.950121	-0.134791
9	7	0	-0.832389	0.800280	-0.027827
10	6	0	-1.934276	2.863740	-0.121044
11	8	0	-2.193556	4.034700	-0.184641
12	6	0	-2.152838	0.631641	-0.030917
13	7	0	-2.839299	1.813239	-0.072030
14	7	0	-2.755449	-0.545335	-0.005146
15	1	0	-2.143472	-1.353155	-0.072040
16	6	0	-4.167856	-0.747569	0.025560
17	6	0	-4.744695	-1.574068	-0.930982
18	6	0	-4.941407	-0.154485	1.016388
19	6	0	-6.113139	-1.799300	-0.897914
20	1	0	-4.124242	-2.028076	-1.690958
21	6	0	-6.313405	-0.369161	1.027158
22	1	0	-4.473100	0.451738	1.779865
23	6	0	-6.899884	-1.191453	0.073797
24	1	0	-6.565215	-2.443079	-1.638568
25	1	0	-6.918291	0.094535	1.792908
26	1	0	-7.966459	-1.363169	0.091069
27	1	0	-3.841978	1.907071	-0.135441
28	1	0	5.552047	1.271698	0.026395
29	1	0	5.044773	3.683413	-0.053650
30	1	0	2.667189	4.416672	-0.112166
31	7	0	2.197202	1.098098	-0.018065
32	6	0	3.714719	-0.775486	0.051671
33	6	0	4.990748	-1.324225	0.071290
34	6	0	5.134815	-2.702127	0.104897
35	1	0	5.866472	-0.697475	0.059391
36	6	0	2.766864	-2.885138	0.096077
37	6	0	4.004911	-3.502726	0.118001
38	1	0	6.121663	-3.141024	0.120012
39	1	0	1.856911	-3.466101	0.105039
40	1	0	4.070831	-4.579106	0.143853
41	7	0	2.625493	-1.559104	0.064154
42	30	0	0.773605	-0.513273	0.024504
43	8	0	-0.453259	-2.344446	-0.078680
44	1	0	-0.413867	-2.811420	-0.922235
45	1	0	-0.525876	-3.003146	0.621295
46	8	0	0.633040	-0.856874	-2.135366
47	1	0	1.445121	-1.013624	-2.629154
48	1	0	0.131976	-0.205792	-2.638631
49	8	0	0.620630	-0.698753	2.167430
50	1	0	-0.222622	-0.546886	2.606547
51	1	0	1.064043	-1.403912	2.650058

Complex E: _805aae_GFP_bipiridil-1H+_Zn2+_+3H2O_M062X_6311++2d2p_PCMw.log

Standard orientation:

Center Number	Atomic Number	Atomic Type	Coordinates (Angstroms) X	Y	Z
1	6	0	4.593039	1.408067	0.003879
2	6	0	4.391266	2.783578	-0.008843
3	6	0	3.107208	3.276190	-0.016613
4	6	0	2.021890	2.386042	-0.012038
5	6	0	3.486865	0.574745	0.007518
6	6	0	0.680606	2.930980	-0.016878
7	6	0	-0.498769	2.281316	-0.013824
8	1	0	0.608187	4.010006	-0.020307
9	7	0	-0.807336	0.945467	-0.010191
10	6	0	-1.821707	3.030039	-0.012210
11	8	0	-1.948002	4.242675	-0.017543
12	6	0	-2.180310	0.923270	-0.005721
13	7	0	-2.817178	2.094223	-0.004748
14	7	0	-2.793635	-0.270292	-0.005817
15	1	0	-2.164087	-1.062732	-0.041120
16	6	0	-4.163932	-0.591132	0.003465
17	6	0	-4.477955	-1.949835	-0.099470
18	6	0	-5.192245	0.344281	0.119492
19	6	0	-5.797449	-2.368452	-0.088809
20	1	0	-3.680055	-2.675897	-0.188585
21	6	0	-6.512109	-0.092374	0.127558
22	1	0	-4.958917	1.391259	0.200642
23	6	0	-6.826817	-1.440178	0.024175
24	1	0	-6.019908	-3.423167	-0.169647
25	1	0	-7.302139	0.640380	0.217863
26	1	0	-7.857381	-1.764511	0.032117
27	1	0	5.593845	1.011365	0.010339
28	1	0	5.237433	3.455254	-0.012177
29	1	0	2.915687	4.338660	-0.025822
30	7	0	2.232691	1.056578	-0.000889
31	6	0	3.625226	-0.910292	0.019885
32	6	0	4.862528	-1.544515	0.031394
33	6	0	4.914891	-2.928475	0.041426
34	1	0	5.777892	-0.976744	0.032849
35	6	0	2.540135	-2.951688	0.026925
36	6	0	3.733305	-3.651483	0.039162
37	1	0	5.870271	-3.432594	0.050819
38	1	0	1.593957	-3.471033	0.023809
39	1	0	3.726815	-4.730217	0.046362
40	7	0	2.485755	-1.618619	0.018206
41	30	0	0.695427	-0.437062	0.015621
42	8	0	-0.604747	-2.248287	-0.122518
43	1	0	-0.604735	-2.668840	-0.990554
44	1	0	-0.728002	-2.935638	0.541039
45	8	0	0.512524	-0.783468	-2.161655
46	1	0	1.310302	-0.934253	-2.679573
47	1	0	0.028120	-0.080523	-2.608352
48	8	0	0.559867	-0.719211	2.167751
49	1	0	-0.285267	-0.517160	2.582887
50	1	0	0.901959	-1.507439	2.601737

Complex-F: _805abe_GFP_bipiridil-1H+_Zn2+_+3H2O_M062X_6311++2d2p_PCMw.log

Standard orientation:

Center Number	Atomic Number	Atomic Type	Coordinates (Angstroms) X	Y	Z
1	6	0	4.528806	1.525096	0.025008
2	6	0	4.274779	2.891197	-0.023127
3	6	0	2.973615	3.334211	-0.076455
4	6	0	1.922346	2.403471	-0.079050
5	6	0	3.454985	0.649921	0.010263
6	6	0	0.562356	2.903152	-0.120761
7	6	0	-0.586423	2.197115	-0.079946
8	1	0	0.450762	3.976077	-0.187799
9	7	0	-0.802852	0.853317	0.013083
10	6	0	-1.925470	2.883211	-0.155039
11	8	0	-2.175074	4.060823	-0.252080
12	6	0	-2.146250	0.627751	-0.015327
13	7	0	-2.821251	1.845443	-0.095169
14	7	0	-2.666465	-0.550899	-0.005047
15	6	0	-4.062692	-0.738201	0.026528
16	6	0	-4.634801	-1.631747	-0.881197
17	6	0	-4.879116	-0.120720	0.977999
18	6	0	-5.999442	-1.878017	-0.857050
19	1	0	-3.996478	-2.122463	-1.603258
20	6	0	-6.245356	-0.371359	0.996019
21	1	0	-4.437384	0.535163	1.716691
22	6	0	-6.812644	-1.246110	0.078007
23	1	0	-6.429491	-2.567095	-1.570689
24	1	0	-6.864522	0.112703	1.738542
25	1	0	-7.875321	-1.441113	0.096474
26	1	0	5.543513	1.167641	0.072798
27	1	0	5.094148	3.595340	-0.016315
28	1	0	2.743513	4.388543	-0.112283
29	7	0	2.183956	1.082554	-0.042945
30	6	0	3.651894	-0.830386	0.048647
31	6	0	4.910528	-1.421376	0.067230
32	6	0	5.008472	-2.803214	0.091950
33	1	0	5.806262	-0.822985	0.059511
34	6	0	2.636095	-2.911391	0.069577
35	6	0	3.853154	-3.569010	0.091367
36	1	0	5.980558	-3.274248	0.107369
37	1	0	1.702696	-3.457329	0.057840
38	1	0	3.886534	-4.647208	0.105112
39	7	0	2.542151	-1.581590	0.054061
40	30	0	0.711993	-0.506760	0.008831
41	8	0	-0.603327	-2.198160	-0.055689
42	1	0	-1.489327	-1.704643	-0.036351
43	1	0	-0.598770	-2.769973	0.717998
44	8	0	0.593437	-0.824671	-2.193835
45	1	0	1.386783	-0.686806	-2.721268
46	1	0	0.324792	-1.737196	-2.348372
47	8	0	0.723163	-0.589729	2.234014
48	1	0	-0.130870	-0.425618	2.647465
49	1	0	1.100612	-1.350683	2.686877
50	1	0	-3.821180	1.945243	-0.170291
